# Different associations of atherogenic index of plasma, triglyceride glucose index, and hemoglobin A1C levels with the risk of coronary artery calcification progression according to established diabetes

**DOI:** 10.1186/s12933-024-02508-4

**Published:** 2024-11-19

**Authors:** Ki-Bum Won, Su-Yeon Choi, Eun Ju Chun, Sung Hak Park, Jidong Sung, Hae Ok Jung, Hyuk-Jae Chang

**Affiliations:** 1 Division of Cardiology, Chung-Ang University Gwangmyeong Medical Center, Chung-Ang University College of Medicine, Gwangmyeong, South Korea; 2 Division of Cardiology, Healthcare System Gangnam Center, Seoul National University Hospital, Seoul, South Korea; 3 Division of Radiology, Seoul National University Bundang Hospital, Seongnam, South Korea; 4 Division of Radiology, Gangnam Heartscan Clinic, Seoul, South Korea; 5 Division of Cardiology, Samsung Medical Center, Heart Stroke & Vascular Institute, Seoul, South Korea; 6 Division of Cardiology, Seoul St. Mary’s Hospital, College of Medicine, The Catholic University of Korea, Seoul, South Korea; 7 Division of Cardiology, Yonsei Cardiovascular Center, Yonsei University Health System, Seoul, South Korea; 8 Division of Cardiology, Severance Cardiovascular Hospital Yonsei-Cedars-Sinai Integrative Cardiovascular Imaging Research Center, Yonsei University College of Medicine, Yonsei University Health System, 50-1 Yonsei-ro, Seodaemun-gu, Seoul, 03722 South Korea

**Keywords:** Atherogenic index of plasma, Triglyceride glucose index, Hemoglobin A1C, Coronary artery calcium score, Diabetes mellitus

## Abstract

**Background:**

Both insulin resistance and hyperglycemia are important risk factors for atherosclerosis. While the characteristics of atherosclerosis are obviously different according to established diabetes, little has been known regarding the risk of coronary artery calcification (CAC) progression related to the biomarkers of atherogenic index of plasma (AIP), triglyceride glucose (TyG) index, and hemoglobin A1C (HbA1C) in conditions with and without diabetes.

**Methods:**

We analyzed 12,326 asymptomatic Korean adults (mean age 51.7 ± 8.5 years; 84.2% males; 15.8% with diabetes) over a median follow-up period of 3.0 years. AIP was defined as the base-10 logarithm of the ratio of triglyceride concentration (mmol/L) to high-density lipoprotein cholesterol (mmol/L). The TyG index was calculated as ln (fasting triglycerides [mg/dL] × fasting glucose [mg/ dL]/2). CAC progression was defined using the SQRT method, as a difference of ≥ 2.5 between the square roots (√) of baseline and follow-up coronary artery calcium scores (CACS) (Δ√transformed CACS). Logistic regression models adjusted for interscan periods were used to estimate the odds ratio (OR).

**Results:**

The levels of AIP, TyG index, and HbA1C were significantly higher in diabetics than in non-diabetics. CAC progression was more frequently observed in diabetics (46.9%) than in non-diabetics (28.0%). After adjusting for age, sex, hypertension, hyperlipidemia, obesity, current smoking status, serum creatinine levels, baseline CACS, and interscan period, AIP (per-0.1 unit increase) was associated with CAC progression in only non-diabetics (OR: 1.04, 95% confidence interval [CI]: 1.02 − 1.06; *P* < 0.001). In contrast, HbA1C level (per-1% increase) was significantly associated with CAC progression in only diabetics (OR: 1.19, 95% CI: 1.08 − 1.32; *P* = 0.001). The TyG index (per-1 unit increase) was associated with CAC progression in both non-diabetics (OR: 1.32, 95% CI: 1.19 − 1.46; *P* < 0.001) and diabetics (OR: 1.33, 95% CI: 1.10 − 1.60; *P* = 0.003).

**Conclusions:**

The associations between AIP, TyG index, and HbA1C levels with CAC progression vary according to established diabetes. Of these biomarkers, TyG index is independently associated with CAC progression irrespective of established diabetes.

**Graphical abstract:**

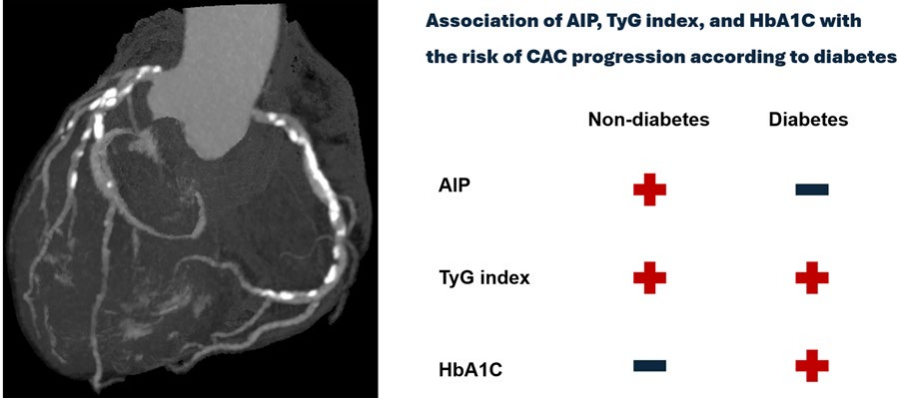

**Supplementary Information:**

The online version contains supplementary material available at 10.1186/s12933-024-02508-4.

## Background

Clinical conditions with insulin resistance (IR), such as obesity, metabolic syndrome, and diabetes, are significantly associated with dyslipidemia, typically presenting as elevated triglyceride and reduced high-density lipoprotein cholesterol (HDL-C) levels [1]. Recently, the atherogenic index of plasma (AIP), a novel biomarker based on the ratio of triglyceride and HDL-C levels, is known to have a close relationship with IR-related metabolic disease [2–5]. Similarly, the triglyceride glucose (TyG) index has emerged as a more practical and reliable predictor of IR compared to the traditional methods including the hyperinsulinemic-euglycemic clamp test and the homeostasis model assessment of IR index [6, 7]. Additionally, hemoglobin A1C (HbA1C) has been proposed as a useful biomarker for improving cardiovascular (CV) risk assessment, given that diabetes is a well-established risk factor for CV disease [8].

Characteristics of atherosclerotic CV disease differ significantly based on the presence of established diabetes [9, 10]. Recent studies have demonstrated significant associations of AIP [11, 12], TyG index [13, 14], and HbA1C [15, 16] levels with subclinical atherosclerosis. However, data on the association between these biomarkers and changes of coronary atherosclerosis focusing on the presence of established diabetes are limited. In asymptomatic adult populations, the coronary artery calcium score (CACS) has been widely used to stratify the risk of major adverse events, providing additional prognostic values beyond traditional CV risk factors [17–19]. We aimed to evaluate the association of AIP, TyG index, and HbA1C levels with the risk of coronary artery calcification (CAC) progression in asymptomatic Korean adults with and without established diabetes.

## Methods

### Study design and participants

We analyzed the data from the Korea Initiatives on Coronary Artery Calcification (KOICA) registry, a single-ethnicity, multicenter, observational study designed to evaluate the effectiveness of CACS for the primary prevention of CV disease in asymptomatic Korean adults [20]. Data were obtained from the databases of six healthcare centers in South Korea (Severance Cardiovascular Hospital, Samsung Medical Center, Seoul St. Mary’s Hospital, Seoul National University Hospital, Seoul National University Bundang Hospital, and Gangnam Heartscan Clinic). In brief, this study included 12,326 subjects who underwent at least two CAC scan examinations, with available data on the AIP, TyG index, HbA1C, and diabetes, from December 2003 to August 2017. A flowchart of the present study is presented in Additional file 1: Figure S1. The data were obtained during visits to each healthcare center. Self-reported medical questionnaires were used to collect information on participants’ medical history. Weight and height were measured with participants wearing light clothing and no shoes, and body mass index (BMI) was calculated as weight (kg) divided by height squared (m^2^). Blood pressure was measured using an automatic manometer on the right arm after a minimum of 5 min of rest. Blood samples were collected after at least 8 h of fasting to assess total cholesterol, triglycerides, HDL-C, low-density lipoprotein cholesterol (LDL-C), creatinine, glucose, and HbA1C levels. Hypertension was defined as a systolic blood pressure (SBP) ≥ 140 mmHg, diastolic blood pressure (DBP) ≥ 90 mmHg, a previous diagnosis of hypertension, or use of anti-hypertensive medication. Diabetes was defined as a fasting glucose level ≥ 126 mg/dL, HbA1C level ≥ 6.5%, a prior diagnosis of diabetes, or use of anti-diabetic medication. Hyperlipidemia was defined as a total cholesterol level ≥ 240 mg/dL or the use of anti-hyperlipidemic medication. Obesity was classified as a BMI ≥ 25.0 kg/m^2^, based on the cutoffs for the Korean population. Current smoking status was recorded for participants who either smoked at the time of the study or had quit < 1 month prior. The AIP was calculated as the base-10 logarithm of the ratio of triglycerides (mmol/L) to HDL-C (mmol/L) [21], while the TyG index was determined as ln (triglycerides [mg/dL] × glucose [mg/dL] / 2) [22]. CACS was measured according to the Agatston scoring system [23]. CAC progression was defined using the SQRT method, specifically as a difference of ≥ 2.5 between the square roots (√) of the baseline and follow-up CACS values (Δ√transformed CACS) [24, 25]. CACS was evaluated using > 16-slice multi-detector computed tomography (CT) scanners (Siemens 16-slice Sensation [Siemens, Forchheim, Germany], Philips Brilliance 256 iCT [Philips Healthcare, Cleveland, OH], Philips Brilliance 40-channel multi-detector CT [Philips Healthcare], and GE 64-slice Lightspeed [GE Healthcare, Milwaukee, WI]). All CAC scans were performed using a scan protocol of standard ECG-triggering methods. All laboratory examination and image acquisition methods adhered to the relevant guidelines and regulations. The study protocol was approved by the Institutional Review Board Committees of Severance Cardiovascular Hospital (IRB No: 4-2014-0309).

### Statistical analysis

Continuous variables are presented as means ± standard deviations or median (interquartile range), while categorical variables are expressed as absolute values and percentages. Comparisons of continuous variables between two groups were performed using the independent *t*-test or Mann–Whitney U test, as appropriate. Categorical variables were compared using the χ^2^-test or Fisher’s exact test, as appropriate. Logistic regression model with adjustment of interscan periods was conducted to evaluate the association between individual clinical variable and the risk of CAC progression. Multivariate logistic regression analysis was used to identify the independent associations of the AIP, TyG index, and HbA1C level with the risk of CAC progression, adjusting for age, sex, hypertension, hyperlipidemia, obesity, current smoking status, serum creatinine levels, baseline CACS, and interscan intervals; the forced entry method was used to enter independent variables into the multivariate logistic regression model. All statistical analyses were performed using the Statistical Package for the Social Sciences (SPSS) version 19 (Chicago, IL) and SAS version 9.1.3 (SAS Institute Inc., Cary, NC). A P-value of < 0.05 was considered statistically significant for all analyses.

## Results

### Baseline characteristics

The mean age of the participants was 51.7 ± 8.5 years, with 10,382 (84.2%) being male. The median follow-up period was 3.0 (2.0 − 5.0) years. Table 1 summarizes the baseline characteristics according to diabetic status. The prevalence of established diabetes was 13.8%. Traditional risk factors, including male sex, hypertension, hyperlipidemia, obesity, and current smoking, were more prevalent in subjects with diabetes than in those without diabetes. Compared with non-diabetics, diabetics had higher levels of triglycerides, glucose, AIP, TyG index, and HbA1C but had lower levels of HDL-C and LDL-C (all *P* < 0.05).

**Table 1:** Baseline characteristics

	Overall (*n* = 12326)	Non-diabetes (*n* = 10623)	Diabetes (*n* = 1703)	*P*
Age, years	51.7 ± 8.5	51.1 ± 8.3	55.3 ± 8.5	< 0.001
Male, n (%)	10,382 (84.2)	8868 (83.5)	1514 (88.9)	< 0.001
SBP, mmHg	119.6 ± 15.0	119.1 ± 14.9	122.4 ± 15.7	< 0.001
DBP, mmHg	75.0 ± 10.5	74.9 ± 10.6	76.2 ± 10.4	< 0.001
BMI, kg/m^2^	24.6 ± 2.8	24.4 ± 2.7	25.2 ± 2.9	< 0.001
Hypertension, n (%)	4016 (33.6)	3098 (30.1)	918 (55.2)	< 0.001
Hyperlipidemia, n (%)	3455 (28.0)	2759 (26.0)	696 (40.9)	< 0.001
Obesity, n (%)	4314 (42.2)	864 (40.8)	5178 (50.9)	< 0.001
Current smoking, n (%)	3229 (28.5)	2741 (28.1)	488 (31.2)	0.012
Laboratory findings				
Total cholesterol, mg/dL	197.5 ± 34.0	198.7 ± 33.5	189.6 ± 36.5	< 0.001
Triglycerides, mg/dL	141.7 ± 89.4	139.3 ± 87.1	156.3 ± 101.7	< 0.001
HDL-C, mg/dL	53.3 ± 16.0	53.7 ± 15.8	51.0 ± 16.5	< 0.001
LDL-C, mg/dL	122.0 ± 31.7	123.2 ± 31.4	114.6 ± 32.9	< 0.001
Glucose, mg/dL	97.8 ± 20.3	92.8 ± 10.1	128.9 ± 35.1	< 0.001
Creatinine, mg/dL	0.95 ± 0.17	0.95 ± 0.17	0.95 ± 0.20	0.722
AIP, unit	0.01 (-0.19, 0.21)	-0.01 (-0.20, 0.20)	0.08 (-0.11, 0.27)	< 0.001
TyG index, unit	8.7 ± 0.6	8.6 ± 0.6	9.0 ± 0.6	< 0.001
HbA1C, %	5.68 ± 0.74	5.47 ± 0.36	6.83 ± 1.14	< 0.001

Values are given as mean ± standard deviation, median (interquartile range), or number (%)

*BMI* body mass index, *DBP* diastolic blood pressure, *DM* diabetes mellitus, *HDL-C* high-density lipoprotein cholesterol, *LDL-C* low-density lipoprotein cholesterol, *SBP* systolic blood pressure

### Baseline and changes of CAC according to diabetic status

**Fig. 1:**
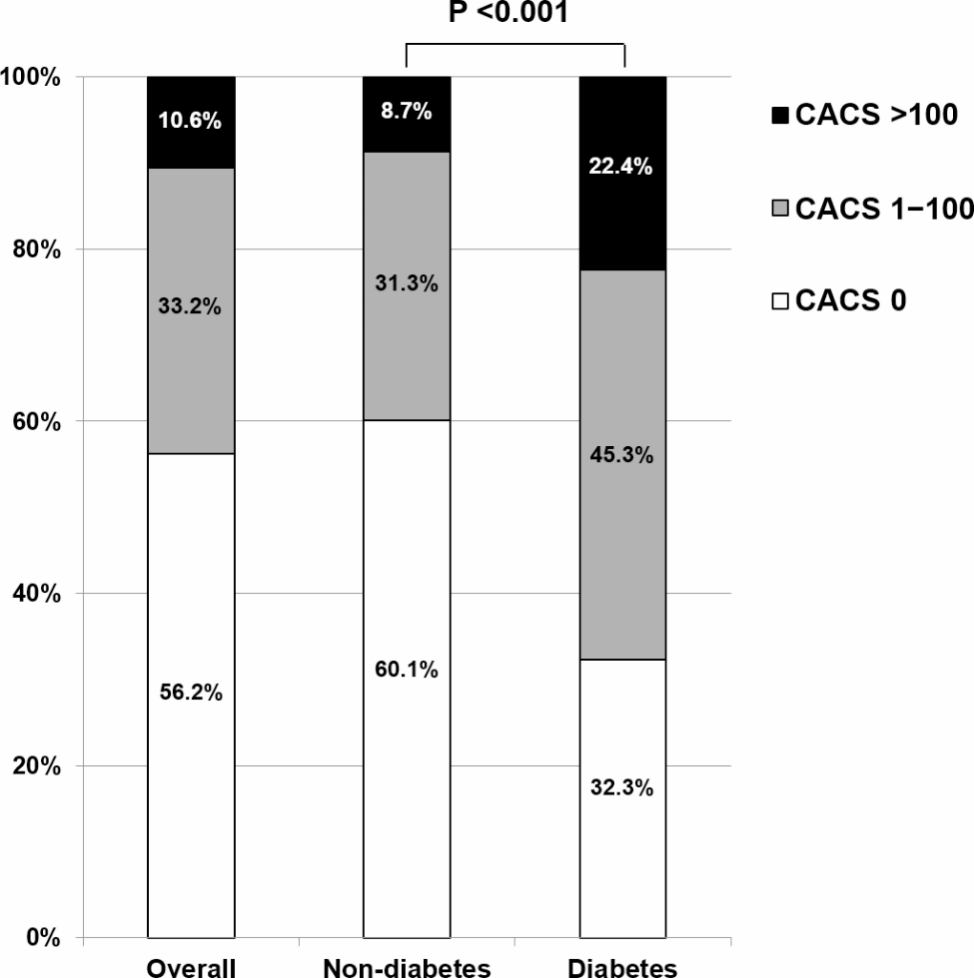
Comparison of baseline CACS according to diabetic status. *CACS* coronary artery calcium score

**Fig. 2:**
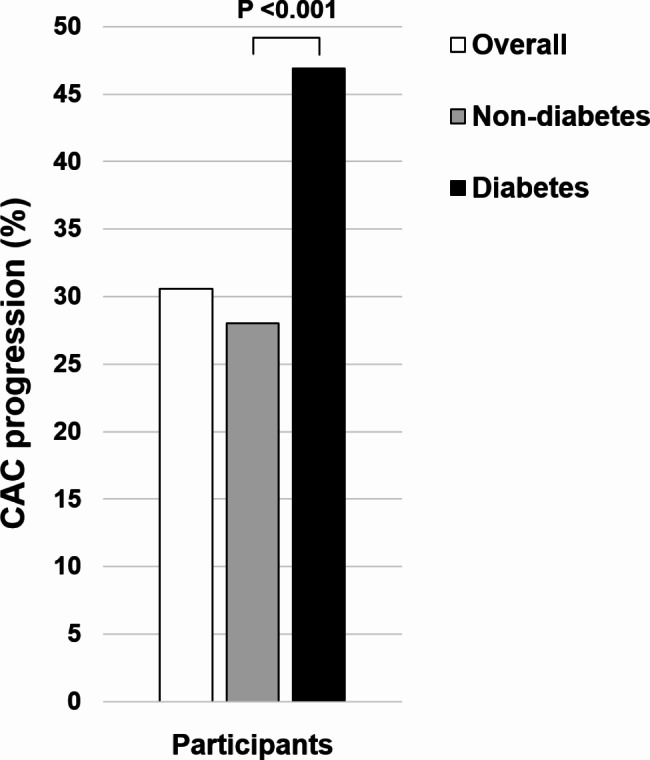
Incidence of CAC progression according to diabetic status. *CAC* coronary artery calcification

At baseline, the categorical CACS distribution differed significantly between non-diabetics and diabetics. Among non-diabetics, 60.1%, 31.3%, and 8.7% had a CACS of 0, between 1 and 100, and > 100, respectively. In contrast, among diabetics, 32.3%, 45.3%, and 22.4% had a CACS of 0, between 1 and 100, and > 100, respectively (*P* < 0.001) (Fig. 1). Compared to non-diabetics, the incidence of CAC progression was higher in diabetics during the follow-up period (28.0 vs. 46.9%, *P* < 0.001) (Fig. 2). The incidence of CAC progression according to baseline categorical CACS in non-diabetics and diabetics is present in Additional file 2: Table S1.

### Association between clinical factors and CAC progression according to diabetic status

Age, male sex, hypertension, obesity, TyG index, HbA1C, and baseline CACS were significantly and positively associated with the risk of CAC progression in both non-diabetics and diabetics. Hyperlipidemia and AIP were associated with an increased risk of CAC progression in only non-diabetics (all *P* < 0.05) (Table 2). Results of restricted cubic spines analysis regarding the association of AIP, TyG index, and HbA1C with the risk of CAC progression in non-diabetics and diabetics are present in Additional file 3: Figure S2.

**Table 2:** Individual clinical factors and the risk of CAC progression

	CAC progression					
	Overall		Non-diabetes		Diabetes	
	OR (95% CI)	P	OR (95% CI)	P	OR (95% CI)	P
Age (per-1 year increase)	1.09 (1.08 − 1.09)	< 0.001	1.09 (1.08 − 1.10)	< 0.001	1.05 (1.04 − 1.06)	< 0.001
Male sex	2.50 (2.19 − 2.86)	< 0.001	2.57 (2.22 − 2.98)	< 0.001	1.78 (1.28 − 2.48)	0.001
Hypertension	2.60 (2.38 − 2.84)	< 0.001	2.66 (2.41 − 2.94)	< 0.001	1.35 (1.09 − 1.66)	0.005
Hyperlipidemia	1.68 (1.54 − 1.83)	< 0.001	1.72 (1.56 − 1.89)	< 0.001	1.02 (0.83 − 1.25)	0.839
Obesity	1.55 (1.43 − 1.68)	< 0.001	1.54 (1.41 − 1.69)	< 0.001	1.25 (1.02 − 1.53)	0.031
Current smoking	1.06 (0.96 − 1.16)	0.260	1.10 (0.99 − 1.22)	0.079	0.89 (0.63 − 1.08)	0.059
AIP (per-0.1 unit increase)	1.08 (1.07 − 1.10)	< 0.001	1.08 (1.07 − 1.10)	< 0.001	1.03 (1.00 − 1.07)	0.073
TyG index (per-1 unit increase)	1.73 (1.61 − 1.86)	< 0.001	1.63 (1.50 − 1.76)	< 0.001	1.20 (1.02 − 1.41)	0.031
HbA1C (per-1% increase)	1.51 (1.42 − 1.60)	< 0.001	1.42 (1.24 − 1.61)	< 0.001	1.13 (1.03 − 1.23)	0.008
Baseline CACS (per-10 unit increase)	1.03 (1.03 − 1.04)	< 0.001	1.04 (1.03 − 1.04)	< 0.001	1.01 (1.01 − 1.02)	< 0.001

Analysis was adjusted for interscan periods

*AIP* atherogenic index of plasma, *CAC* coronary artery calcification, *CACS* coronary artery calcium score, *CI* confidence interval, *HbA1C* hemoglobin A1C, *OR* odds ratio, *TyG* triglyceride glucose

### Risk of CAC progression related to AIP, TyG index, and HbA1C level according to diabetic status

After adjusting for age, sex, hypertension, hyperlipidemia, obesity, current smoking status, serum creatinine levels, baseline CACS, and interscan periods, AIP (per-0.1 unit increase) was significantly associated with CAC progression in only non-diabetics (odds ratio [OR]: 1.04, 95% confidence interval [CI]: 1.02 − 1.06; *P* < 0.001). In contrast, HbA1C levels (per-1% increase) were significantly associated with CAC progression in only diabetics (OR: 1.19, 95% CI: 1.08 − 1.32; *P* = 0.001). The TyG index (per-1 unit increase) was associated with an increased risk of CAC progression in both non-diabetics (OR: 1.32, 95% CI: 1.19 − 1.46; *P* < 0.001) and diabetics (OR: 1.33, 95% CI: 1.10 − 1.60; *P* = 0.003) (Table 3). Results of the subgroup analysis with respect to the association of AIP, TyG index, and HbA1C level with the risk of CAC progression in non-diabetics and diabetics are present in Additional file 4: Table S2.

**Table 3:** The risk of CAC progression related to AIP, TyG index, and HbA1C level according to diabetic status

	CAC progression					
	Overall		Non-diabetes		Diabetes	
	OR (95% CI)	P	OR (95% CI)	P	OR (95% CI)	P
AIP (per-0.1 unit increase)	1.04 (1.03 − 1.06)	< 0.001	1.04 (1.02 − 1.06)	< 0.001	1.03 (0.99 − 1.08)	0.100
TyG index (per-1 unit increase)	1.42 (1.31 − 1.55)	< 0.001	1.32 (1.19 − 1.46)	< 0.001	1.33 (1.10 − 1.60)	0.003
HbA1C (per-1% increase)	1.25 (1.18 − 1.34)	< 0.001	0.99 (0.85 − 1.15)	0.850	1.19 (1.08 − 1.32)	0.001

P values for interaction of diabetes with AIP, TyG index, and HbA1C level were 0.033, 0.006, and 0.543, respectively

Analysis was adjusted for age, sex, hypertension, hyperlipidemia, obesity, current smoking, serum creatinine level, baseline CACS, and interscan periods

*AIP* atherogenic index of plasma, *CAC* coronary artery calcification, *CACS* coronary artery calcium score, *CI* confidence interval, *OR* odds ratio, *HbA1C* hemoglobin A1C, *TyG* triglyceride glucose

## Discussion

To the best of our knowledge, the present study is the first large cohort study in East Asia to identify the different associations between AIP, TyG index, and HbA1C levels and the risk of CAC progression according to the clinical condition of established diabetes. In this cohort study with a median follow-up of 3.0 years conducted in South Korea, distinct associations of the AIP, TyG index, and HbA1C level with the risk of CAC progression were observed in asymptomatic adults with and without established diabetes. As is well-known, the levels of AIP, TyG index, and HbA1C, baseline CACS, and the incidence of CAC progression were significantly higher in diabetics compared to non-diabetics. Beyond traditional CV risk factors and baseline CACS, AIP had a positive association with CAC progression in non-diabetics; however, HbA1C level was related to the increased risk of CAC progression in diabetics. Notably, elevated TyG index levels were independently linked to an increased risk of CAC progression irrespective of diabetic status.

The strength of this study is that the risk of CAC progression was assessed in an asymptomatic adult population without severe CAC at baseline. The proportion of CACS > 400 in overall participants was only 2.6%; the prevalence of CACS > 400 in non-diabetics and diabetics was 1.9% and 7.0%, respectively. According to data from the HNR (Heinz Nixdorf Recall) study [26], repeat computed tomography scans after 5 years could provide individual risk readjustment attributable to the increased risk in cases where baseline CACS < 400. However, although a high coronary and CV risk was present in patients with a baseline CACS > 400, additional CACS evaluation did not add prognostic value.

Numerous studies have suggested that IR plays a substantial role in the development of atherosclerotic CV disease [27–29]. AIP has been recognized as a useful marker of IR-related dyslipidemia [1]. Dobiásová et al. previously suggested the AIP as a better lipid marker of plasma atherogenicity [21]. In addition to individual cholesterol levels, AIP showed a positive relation with cholesterol esterification rates, lipoprotein particle size, and remnant lipoproteinemia [21, 30]. Similarly, the TyG index is also considered as a practical marker of IR due to its high sensitivity and specificity, along with its effectiveness and simplicity of measurement [7, 22, 31]. In patients with established diabetes, numerous studies have demonstrated the beneficial effects of glycemic control, as reflected by HbA1C levels, on subclinical coronary atherosclerosis and adverse CV events [15, 32]. The characteristics of subclinical atherosclerosis related to the metabolic abnormalities are somewhat different in non-diabetics and diabetics [10]. However, despite the potential role of CACS in primary CV prevention, data on the changes in CACS associated with biomarkers of AIP, TyG index, and HbA1C according to diabetic status have been limited.

Regarding atherosclerotic CV disease related to AIP, the Progression of Atherosclerotic Plaque Determined by Computed Tomography Angiography Imaging (PARADIGM) registry demonstrated a significant association between the AIP and the rapid progression of coronary atherosclerosis beyond traditional risk factors, as observed through serial coronary CT angiography findings [12]. In the context of secondary prevention, Wang et al. [33] recently reported that AIP had independent predictive value for adverse clinical outcomes in 1133 patients with acute coronary syndrome who underwent percutaneous coronary intervention (PCI) with LDL-C levels < 70 mg/dL. However, the study defined high AIP levels using a cutoff of 0.11, which is challenging to achieve in clinical practice considering that the AIP value calculated from normal triglyceride (150 mg/dL) and HDL-C (40 mg/dL) levels is 0.21. Additionally, data from the Platelet Function and Genotype-Related Long-Term Prognosis in Drug-Eluting Stent–Treated Patients with Coronary Artery Disease (PTRG-DES) consortium, which included 10,735 patients who underwent successful PCI with drug-eluting stents for obstructive coronary artery disease, showed an inverse association between AIP and the risk of high platelet reactivity in patients without acute myocardial infarction (AMI) during a 3-year follow-up; however, AIP levels were not associated with the risk of adverse clinical outcomes, irrespective of AMI status [34]. Recent clinical evidence strongly supports the effectiveness of AIP in assessing the risk of diabetes development and subclinical atherosclerosis progression. Further investigations are necessary to explore the usefulness of AIP in patients with established diabetes or for secondary prevention.

In contrast, the TyG index has garnered attention for its significance in both primary and secondary CV disease prevention. A previous observational cohort study demonstrated a significant and positive relationship between the TyG index and arterial stiffness, as estimated using brachial-ankle pulse wave velocity, independent of established diabetes [35]. Beyond its value in predicting subclinical atherosclerosis, numerous studies have identified the TyG index as a useful prognostic marker in non-diabetics [36], pre-diabetics [37], and established diabetics [38–40] for secondary prevention.

With respect to biochemical mechanisms, elevated triglyceride levels stimulate the activity of cholesteryl ester transfer proteins, which exchange triglycerides from triglyceride-rich lipoproteins with cholesteryl esters from high- and low-density lipoproteins [41]. Triglyceride enrichment of these particles makes them better substrates for lipolysis by hepatic lipase, leading to high-density lipoprotein catabolism and elimination, and the formation of numerous denser low-density lipoprotein particles. These findings provide a potential explanation for why AIP could be an important biomarker for predicting arteriosclerosis in patients with established diabetes. However, Anand et al. [42] investigated the predictive value of clinical variables, including demographic data, traditional risk factors, glycemic control status, medication use, and biomarkers such as serum high-sensitivity C-reactive protein, interleukin-6, and plasma osteoprotegerin, for CAC progression in 398 asymptomatic patients with type 2 diabetes over a mean follow-up of 2.5 ± 0.4 years. Their study found that only suboptimal glycemic control assessed using HbA1C and baseline CACS was independently associated with the risk of CAC progression. This finding highlights the predominant effect of hyperglycemia on CAC progression in established diabetes as well as the significance of optimal glycemic control in preventing CAC progression among patients with diabetes. In addition, they showed the limited availability of other biomarkers to predict CAC progression in diabetic patients despite their increased risk of CAC progression. The present study reaffirmed the strong association of HbA1C with CAC progression in diabetics and further identified that the TyG index could be an effective biomarker for predicting CAC progression irrespective of established diabetes.

According to recent data from the Progression of AtheRosclerotic PlAque DetermIned by Computed TomoGraphic Angiography Imaging (PARADIGM) registry, the baseline coronary plaque burden is the most important factor when compared with clinical and laboratory factors in identifying patients at risk of rapid plaque progression [43]. This finding emphasizes the significance of the early detection of both the presence and progression of subclinical coronary atherosclerosis, as suggested by an HNR study [26]. With respect to the subgroup analysis of the present study, all biomarkers of AIP, TyG index, and HbA1C did not show a significant association with the risk of CAC progression in elderly and baseline CACS > 100 in both patients without and with diabetes. Recent data from the Coronary Artery Risk Development in Young Adults (CARDIA) registry [44] showed that young adults with higher IR levels were more likely to develop CAC in middle age, including obese subjects. Considering that recent studies have suggested the use of CACS to determine individualized therapeutic goals in diverse clinical situations [45–47], it might be helpful to actively utilize these biomarkers in patients who are young and do not have severe coronary calcification after identifying the presence of established diabetes.

There are some limitations in this study. First, as all participants voluntarily visited healthcare centers for general health examinations, there is a potential for selection bias. Second, due to the observational nature of the study, the use of pharmacological agents was not controlled. Third, there was a lack of information with respect to the duration of diabetes and other diseases. Fourth, different CT scanners were used among the participating centers; however, all participants were examined using the same CT scanner with an identical ECG-triggering method during the initial and follow-up image acquisitions. In addition, CAC progression was defined using the SQRT method, considering interscan variability in the present study. Fifth, we did not perform a variability analysis based on strong evidence regarding the variability and reproducibility of the CACS measurements [48, 49]. Finally, the characteristics of diabetic patients in Asia are notably different from those in other regions [50]. In addition, most of the participants (84.2%) in this study were male. These factors may limit the generalizability of the results. However, this study showed an independent association between TyG index and risk of CAC progression, irrespective of established diabetes in East Asia.

## Conclusions

The associations of AIP, TyG index, and HbA1C levels with CAC progression differ depending on established diabetes. In the current study, AIP was positively associated with the risk of CAC progression in only non-diabetics. Conversely, HbA1C showed a significant association with CAC progression in only diabetics. The TyG index independently associated with the risk of CAC progression in both non-diabetic and diabetic population of asymptomatic Korean adults.

## Electronic supplementary material

Below is the link to the electronic supplementary material.

Supplementary Material 1.

Supplementary Material 2.

Supplementary Material 3.

Supplementary Material 4.

## Data Availability

No datasets were generated or analysed during the current study.

## Abbreviations

AMI: Acute myocardial infarction
AIP: Atherogenic index of plasma
BMI: Body mass index
CV: Cardiovascular
CAC: Coronary artery calcification
CACS: Coronary artery calcium score
CI: Confidence interval
DBP: Diastolic blood pressure
HbA1C: Hemoglobin A1C
HDL-C: High-density lipoprotein cholesterol
IR: Insulin resistance
LCL-C: Low-density lipoprotein cholesterol
OR: Odds ratio
SBP: Systolic blood pressure
TyG: Triglyceride glucose
