# Supplementary figures and images for: Different associations of atherogenic index of plasma, triglyceride glucose index, and hemoglobin A1C levels with the risk of coronary artery calcification progression according to established diabetes
Source: Cardiovasc Diabetol. 2024 Nov 19;23:418. doi: 10.1186/s12933-024-02508-4 (PMC11575153; doi:10.1186/s12933-024-02508-4)

**Additional File 1**

**Fig. S1** Overview of the study population


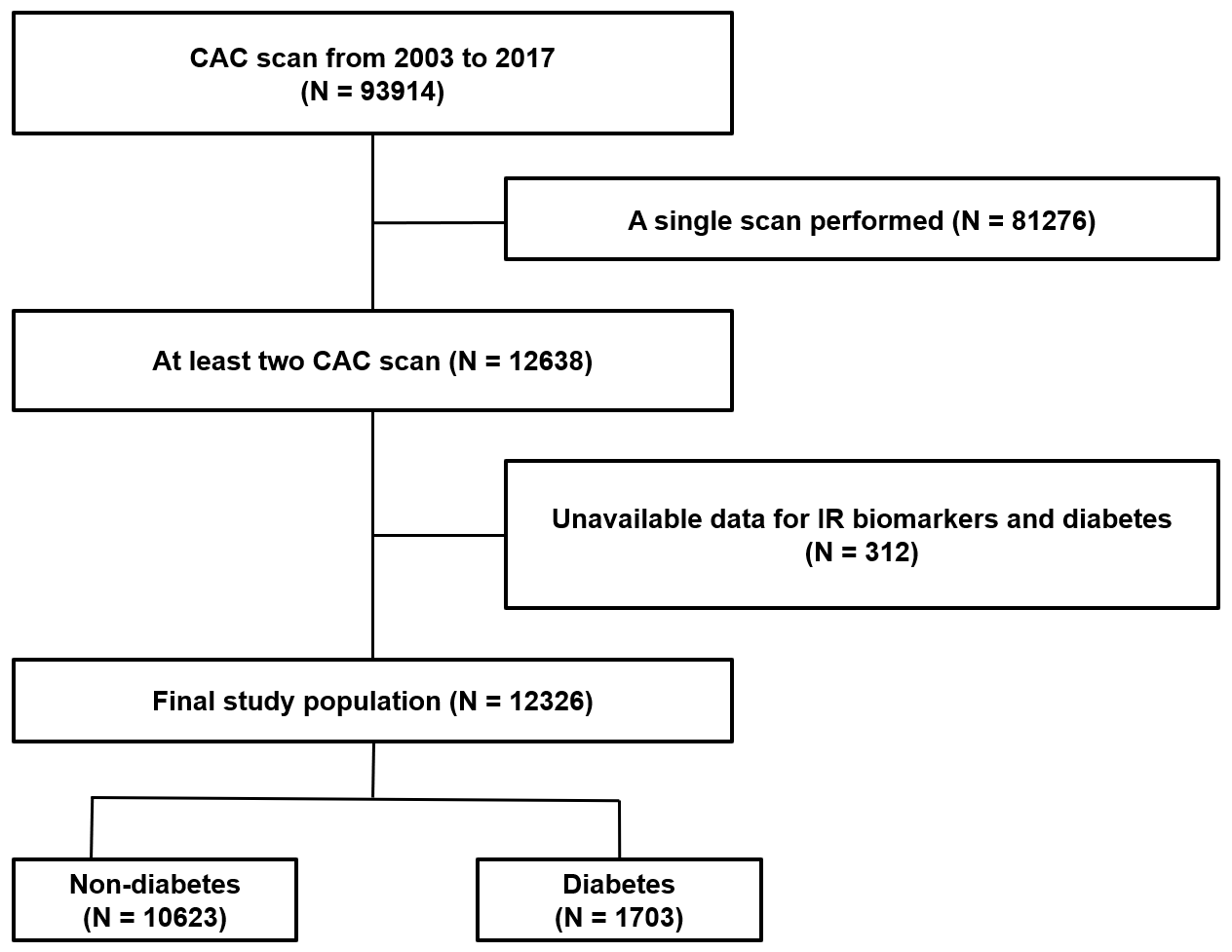

Supplement: Supplementary file 1 — Supplementary Material 1. [file 12933_2024_2508_MOESM1_ESM.docx]
