# Supplementary material for: Different associations of atherogenic index of plasma, triglyceride glucose index, and hemoglobin A1C levels with the risk of coronary artery calcification progression according to established diabetes
Source: Cardiovasc Diabetol. 2024 Nov 19;23:418. doi: 10.1186/s12933-024-02508-4 (PMC11575153; doi:10.1186/s12933-024-02508-4)
Supplement: Supplementary file 2 — Supplementary Material 2. [file 12933_2024_2508_MOESM2_ESM.docx]

**Additional File 2**

**Table S1** Incidence of CAC progression according to baseline CACS in non-diabetes and diabetes

|  | CAC progression | | | |
| --- | --- | --- | --- | --- |
| Non-diabetes  (n = 10623) | CACS 0 (n = 6381) | CACS 1−100 (n = 3322) | CACS >100 (n = 920) | P |
|  | 775 (12.1) | 1733 (52.2) | 462 (50.2) | <0.001 |
| Diabetes  (n = 1703) | CACS 0 (n = 550) | CACS 1−100 (n = 771) | CACS >100 (n = 382) | P |
|  | 125 (22.7) | 454 (58.9) | 220 (57.6) | <0.001 |

Values are given as number (%).

*CAC* coronary artery calcification, *CACS* coronary artery calcium score
