# Supplementary material for: Different associations of atherogenic index of plasma, triglyceride glucose index, and hemoglobin A1C levels with the risk of coronary artery calcification progression according to established diabetes
Source: Cardiovasc Diabetol. 2024 Nov 19;23:418. doi: 10.1186/s12933-024-02508-4 (PMC11575153; doi:10.1186/s12933-024-02508-4)

**Additional File 3**

**Fig. S2** Restricted cubic spines analysis regarding the association of (a) AIP, (b) TyG index, and (c) HbA1C with the risk of CAC progression according to diabetes


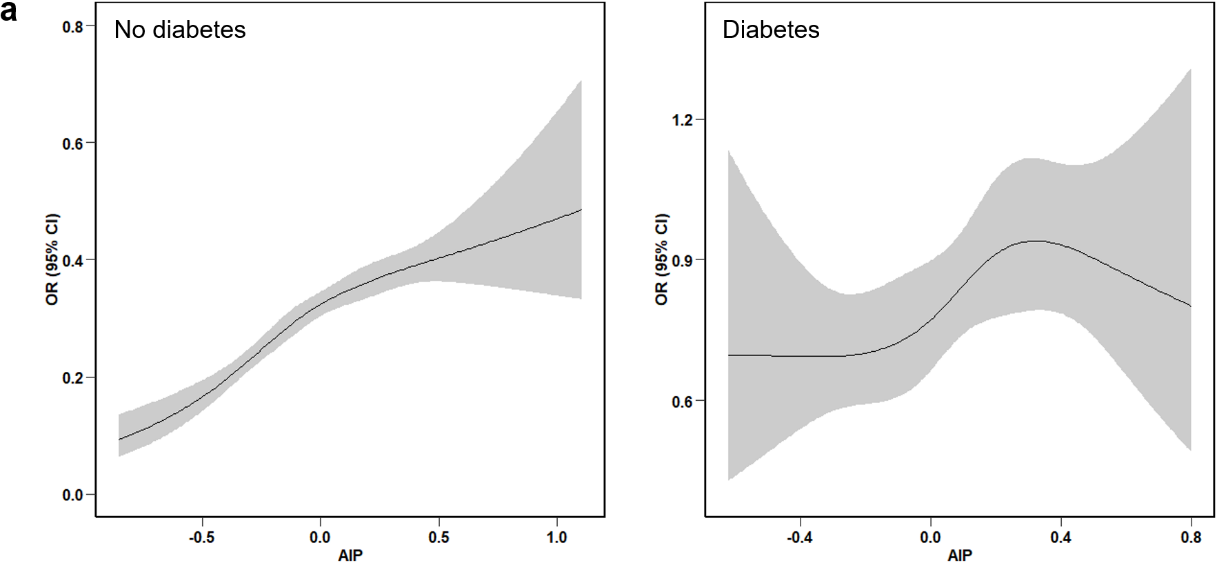


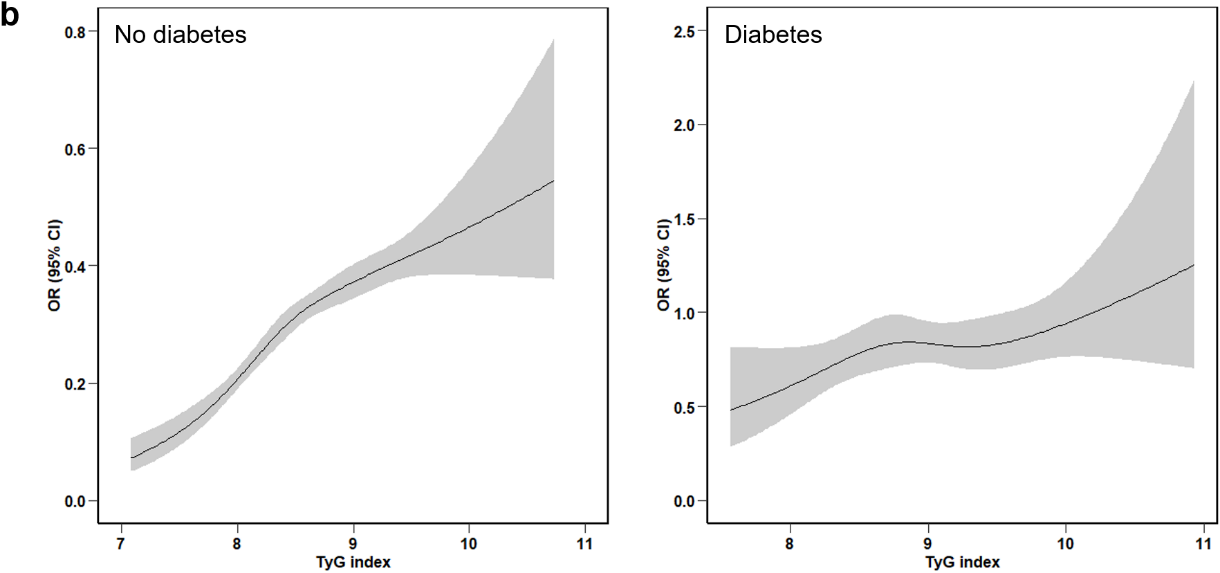


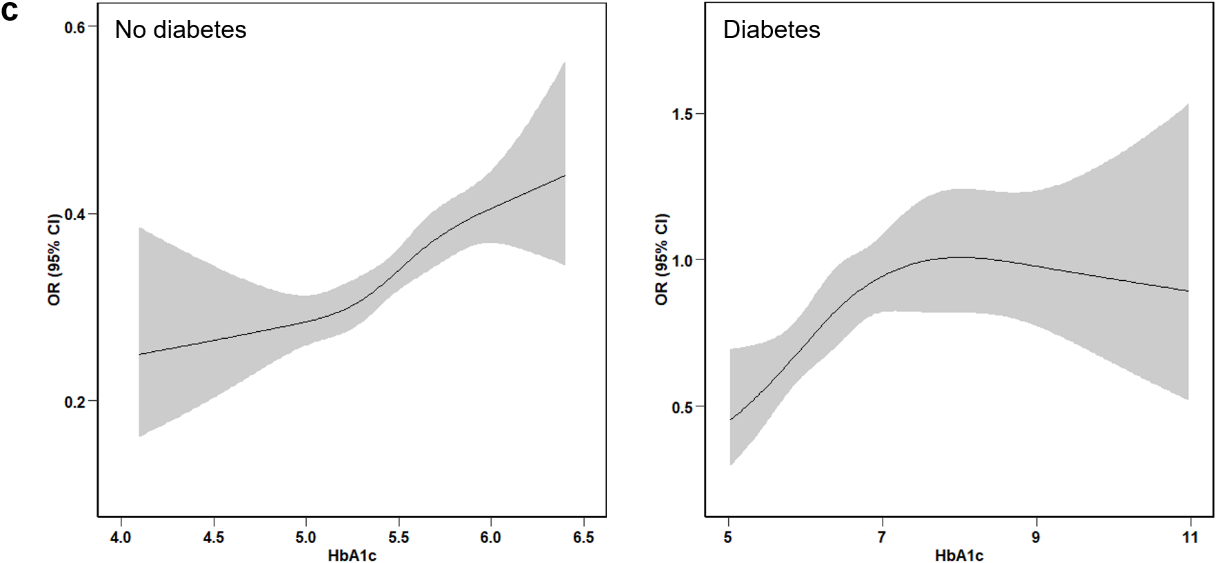

Supplement: Supplementary file 3 — Supplementary Material 3. [file 12933_2024_2508_MOESM3_ESM.docx]
