# Supplementary material for: Different associations of atherogenic index of plasma, triglyceride glucose index, and hemoglobin A1C levels with the risk of coronary artery calcification progression according to established diabetes
Source: Cardiovasc Diabetol. 2024 Nov 19;23:418. doi: 10.1186/s12933-024-02508-4 (PMC11575153; doi:10.1186/s12933-024-02508-4)
Supplement: Supplementary file 4 — Supplementary Material 4. [file 12933_2024_2508_MOESM4_ESM.docx]

**Additional File 4**

**Table S2** Subgroup analysis of the risk of CAC progression related to AIP, TyG index, and HbA1C in non-diabetes and diabetes

|  | Non-diabetes | | P for interaction | Diabetes | | P for interaction |
| --- | --- | --- | --- | --- | --- | --- |
|  | OR (95% CI) | P |  | OR (95% CI) | P |  |
| AIP (per-0.1 unit increase) | | | | | | |
| Age |  |  | <0.001 |  |  | 0.345 |
| <60 years | 1.11 (1.09−1.13) | <0.001 |  | 1.06 (1.01−1.10) | 0.009 |  |
| ≥60 years | 1.01 (0.98−1.06) | 0.479 |  | 1.01 (0.94−1.09) | 0.752 |  |
| Gender |  |  | 0.002 |  |  | 0.638 |
| Female | 1.13 (1.08−1.19) | <0.001 |  | 1.00 (0.90−1.10) | 0.933 |  |
| Male | 1.05 (1.03−1.07) | <0.001 |  | 1.03 (0.99−1.07) | 0.166 |  |
| Hypertension |  |  | 0.003 |  |  | 0.651 |
| No | 1.09 (1.07−1.11) | <0.001 |  | 1.02 (0.97−1.08) | 0.392 |  |
| Yes | 1.04 (1.01−1.07) | 0.007 |  | 1.04 (0.99−1.09) | 0.133 |  |
| Hyperlipidemia |  |  | 0.008 |  |  | 0.961 |
| No | 1.08 (1.06−1.11) | <0.001 |  | 1.03 (0.98−1.08) | 0.193 |  |
| Yes | 1.04 (1.01−1.07) | 0.012 |  | 1.04 (0.98−1.09) | 0.200 |  |
| BMI |  |  | 0.002 |  |  | 0.646 |
| <25.0 kg/m^2^ | 1.09 (1.07−1.11) | <0.001 |  | 1.02 (0.96−1.07) | 0.565 |  |
| ≥25.0 kg/m^2^ | 1.04 (1.01−1.06) | 0.004 |  | 1.03 (0.98−1.09) | 0.194 |  |
| Current smoking |  |  | 0.019 |  |  | 0.146 |
| No | 1.07 (1.05−1.09) | <0.001 |  | 1.01 (0.97−1.06) | 0.532 |  |
| Yes | 1.11 (1.08−1.15) | <0.001 |  | 1.08 (1.01−1.15) | 0.031 |  |
| Baseline CACS |  |  | 0.093 |  |  | 0.794 |
| ≤100 | 1.08 (1.07−1.1) | <0.001 |  | 1.04 (1.00−1.08) | 0.071 |  |
| >100 | 1.03 (0.98−1.09) | 0.200 |  | 1.02 (0.94−1.10) | 0.667 |  |
| TyG index (per-1 unit increase) | | | | | | |
| Age |  |  | <0.001 |  |  | 0.119 |
| <60 years | 1.88 (1.71−2.06) | <0.001 |  | 1.41 (1.16−1.70) | <0.001 |  |
| ≥60 years | 1.13 (0.91−1.39) | 0.270 |  | 1.00 (0.70−1.43) | 0.990 |  |
| Gender |  |  | 0.005 |  |  | 0.825 |
| Female | 2.07 (1.60−2.67) | <0.001 |  | 1.09 (0.67−1.76) | 0.736 |  |
| Male | 1.40 (1.28−1.53) | <0.001 |  | 1.16 (0.98−1.39) | 0.091 |  |
| Hypertension |  |  | 0.002 |  |  | 0.959 |
| No | 1.68 (1.51−1.87) | <0.001 |  | 1.22 (0.96−1.54) | 0.112 |  |
| Yes | 1.27 (1.10−1.47) | 0.001 |  | 1.19 (0.95−1.50) | 0.130 |  |
| Hyperlipidemia |  |  | <0.001 |  |  | 0.841 |
| No | 1.69 (1.53−1.87) | <0.001 |  | 1.18 (0.94−1.47) | 0.159 |  |
| Yes | 1.19 (1.03−1.38) | 0.020 |  | 1.24 (0.98−1.57) | 0.079 |  |
| BMI |  |  | <0.001 |  |  | 0.829 |
| <25.0 kg/m^2^ | 1.76 (1.57−1.98) | <0.001 |  | 1.18 (0.92−1.50) | 0.187 |  |
| ≥25.0 kg/m^2^ | 1.26 (1.11−1.43) | <0.001 |  | 1.15 (0.91−1.44) | 0.236 |  |
| Current smoking |  |  | 0.092 |  |  | 0.549 |
| No | 1.55 (1.40−1.72) | <0.001 |  | 1.18 (0.96−1.45) | 0.122 |  |
| Yes | 1.81 (1.54−2.12) | <0.001 |  | 1.32 (0.96−1.80) | 0.085 |  |
| Baseline CACS |  |  | 0.037 |  |  | 0.393 |
| ≤100 | 1.65 (1.51−1.80) | <0.001 |  | 1.30 (1.08−1.57) | 0.006 |  |
| >100 | 1.21 (0.92−1.60) | 0.178 |  | 1.04 (0.72−1.51) | 0.834 |  |
| HbA1C (per-1 % increase) | | | | | | |
| Age |  |  | <0.001 |  |  | 0.671 |
| <60 years | 1.48 (1.27−1.72) | <0.001 |  | 1.13 (1.03−1.26) | 0.015 |  |
| ≥60 years | 0.76 (0.61−1.10) | 0.090 |  | 1.21 (0.99−1.48) | 0.057 |  |
| Gender |  |  | 0.113 |  |  | 0.846 |
| Female | 2.01 (1.32−3.06) | 0.001 |  | 1.15 (0.88−1.51) | 0.304 |  |
| Male | 1.47 (1.28−1.69) | <0.001 |  | 1.13 (1.03−1.24) | 0.014 |  |
| Hypertension |  |  | 0.384 |  |  | 0.343 |
| No | 1.34 (1.13−1.60) | 0.001 |  | 1.21 (1.06−1.38) | 0.006 |  |
| Yes | 1.21 (0.98−1.51) | 0.083 |  | 1.11 (0.98−1.27) | 0.095 |  |
| Hyperlipidemia |  |  | 0.687 |  |  | 0.644 |
| No | 1.36 (1.16−1.59) | <0.001 |  | 1.11 (0.98−1.26) | 0.094 |  |
| Yes | 1.29 (1.02−1.63) | 0.034 |  | 1.15 (1.01−1.32) | 0.035 |  |
| BMI |  |  | 0.452 |  |  | 0.619 |
| <25.0 kg/m^2^ | 1.40 (1.16−1.68) | <0.001 |  | 1.16 (1.02−1.31) | 0.023 |  |
| ≥25.0 kg/m^2^ | 1.26 (1.04−1.53) | 0.018 |  | 1.10 (0.97−1.25) | 0.150 |  |
| Current smoking |  |  | 0.591 |  |  | 0.295 |
| No | 1.39 (1.18−1.64) | <0.001 |  | 1.20 (1.06−1.36) | 0.003 |  |
| Yes | 1.43 (1.11−1.84) | 0.006 |  | 1.09 (0.93−1.27) | 0.299 |  |
| Baseline CACS |  |  | 0.003 |  |  | 0.113 |
| ≤100 | 1.36 (1.18−1.56) | <0.001 |  | 1.17 (1.05−1.30) | 0.003 |  |
| >100 | 0.75 (0.48−1.16) | 0.192 |  | 1.00 (0.82−1.22) | 0.986 |  |

Analysis was adjusted for interscan periods.

*AIP* atherogenic index of plasma, *BMI* body mass index, *CAC* coronary artery calcification, *CACS* coronary artery calcium score, *CI* confidence interval, *OR* odds ratio, *HbA1C* hemoglobin A1C, *TyG* triglyceride glucose
